# The pivotal roles of neddylation pathway in immunoregulation

**DOI:** 10.1002/iid3.335

**Published:** 2020-08-04

**Authors:** Yun Lu, Xuguang Yang

**Affiliations:** ^1^ Cancer Institute, Department of Oncology, Longhua Hospital Shanghai University of Traditional Chinese Medicine Shanghai China

**Keywords:** immune cells, immune response, immune‐related disease, neddylation, signaling molecule

## Abstract

**Introduction:**

Protein neddylation, one of the most important posttranslational modifications that tagging neuronal precursor cell‐expressed developmentally downregulated protein 8 onto substrate proteins, plays fundamental roles in the process of many cellular functions. A number of studies have demonstrated the critical roles of neddylation modification in multiple pathophysiological processes, but its regulatory role in the immune system has only been finitely unveiled.

**Methods:**

In this review, the latest advances in the field of neddylation modification in regulating the immune responses are succinctly discussed.

**Results:**

Neddylation modification acts as a crucial modulator of innate immune cells (neutrophils, macrophages, and dendritic cells) and lymphocytes. Dysregulation of neddylation alters characteristics and functions of those cells due to abnormal degradation of key signaling molecules involved in immunoregulation. Furthermore, the ectopic immune responses caused by the abnormal neddylation play pivotal roles in a variety of immune‐related diseases, such as infection, inflammation, and cancer.

**Conclusions:**

The pivotal roles of neddylation pathway in immunoregulation are attracted more and more attention, which may provide new insights into the pathogenesis of a variety of immune‐related diseases and help to indicate new therapeutic targets and potential treatment strategies.

**Abbreviations**
ATP, adenosine triphosphate; CCL2: C‐C motif chemokine ligand 2
CRLs: Cullin‐RING E3 ligases
CSN: COP9 signalosome
DAMPs: damage associated molecular patterns
DCN1: defective in Cullin neddylation 1
DCs: dendritic cells
Deptor: Dep domain containing mTOR interacting protein
Erk: extracellular regulated protein kinases
FoxO1: forkhead box O1
HIF‐1: hypoxia‐inducible factor 1
Hu‐antigen R: HuR
HUVECs: human umbilical vein endothelial cells
ICAM: intercellular cell adhesion molecule
IFN‐β: interferon‐β
IFN‐γ: interferon‐1γ
IL‐10: interleukin‐10
IL‐1β: interleukin‐1β
IL‐6: interleukin‐6
Itch: itchy E3 ubiquitin‐protein ligase
IκB: inhibitor of nuclear factor kappa B
LPS: lipopolysaccharide
MDM2: murine double minute 2
mTOR: mechanistic target of rapamycin kinase
NAE1: NEDD8‐activating enzyme E1 subunit 1
NEDD8: neuronal precursor cell‐expressed developmentally downregulated protein 8
NF‐κB: nuclear factor kappa B
OVA, ovalbumin; PAMPs: pathogen‐associated molecular patterns
PD‐1: programmed cell death protein 1; PD‐L1
RBX1/2: RING‐box protein ½
RING: really interesting new gene
SAG: sensitive to apoptosis gene
SENP8: SUMO peptidase family member, NEDD8 specific
SKP‐1: S‐phase kinase‐associated protein 1
ssRNA: single‐stranded ribonucleic acid
TAMs: tumor‐associated macrophages
Tfh: follicular helper T
Th: T helper
TNF‐α: tumor necrosis factor‐α
Tregs: regulatory T Cells
UBA3: ubiquitin‐like modifier activating enzyme 3
UBE2F: ubiquitin conjugating enzyme E2 F
UBE2M: ubiquitin conjugating enzyme E2 M
VHL: von Hippel‐Lindau protein
βTrCP: β‐transducin repeat‐containing protein

## 1 INTRODUCTION

Protein neddylation is the posttranslational modification that conjugating an ubiquitin‐like molecule neuronal precursor cell‐expressed developmentally downregulated protein 8 (NEDD8) to the lysine residue in substrate proteins via a successive three‐step enzymatic reaction.^1^, ^2^, ^3^ Analogous to ubiquitylation, the exposed C‐terminal glycine of NEDD8 is activated by NEDD8‐activating enzyme E1, a heterodimer composed of NEDD8‐activating enzyme E1 subunit 1 (also known as APPBP1) and ubiquitin‐like modifier activating enzyme 3 (UBA3) (also known as NAEβ), in an ATP‐dependent manner.^4^ Then, activated NEDD8 is transferred to NEDD8‐conjugating enzyme E2, UBE2M (also known as UBC12) or UBE2F, via forming a thiolester linkage.^5^ The substrate‐specific NEDD8‐E3 ligases subsequently transfer NEDD8 to substrate protein forming an isopeptide bond. Unlike E3 ubiquitin ligases, a limited number of NEDD8‐E3 ligases is identified. Most of them, such as really interesting new gene (RING)‐box protein 1 (RBX1) (also known as ROC1), RBX2 (also known as ROC2) and murine double minute 2, have the RING domain structure.^6^ DCN1 is the sole exception that does not contain a RING domain for its catalytic activity.^7^, ^8^ Cullin‐RING E3 ligases (CRLs) represent the largest superfamily of multisubunit E3s as well as the best‐characterized substrates of neddylation. CRLs activation requires neddylation of Cullin proteins to facilitate conformational change of CRLs and subsequent substrate ubiquitination.^9^ Besides of Cullins, several non‐Cullin proteins (eg, von Hippel‐Lindau protein [VHL], Hu‐antigen R) have also been identified as the substrates of neddylation.^10^, ^11^ Neddylation is a reversible modification of substrates and can be reversed by the action of deneddylating enzymes including COP9 Signalosome (CSN), SENP8 etc.^12^, ^13^ CSN mainly deneddylates Cullin proteins and ectopic CSN expression may affect the stability of CRLs.^14^

Neddylation modification is of vital importance in biological processes and its dysregulation causes abnormal degradation of their crucial substrate proteins along with diseases. MLN4924 (known as pevonedistat) is a first‐in‐class small molecular inhibitor of UBA3 currently in phase I/II clinical trials for patients suffering from cancer, capable of blocking the entire neddylation modification.^15^ MLN4924 covalently adducts with NEDD8 and competitively binds to the active site of NAEβ, effectively blocks CRLs activation. Numerous investigations have revealed that inactivation of CRLs triggers multiple biological events including cell cycle arrest, apoptosis, senescence, autophagy, angiogenesis, ciliogenesis, mitochondrial morphology etc.^16^, ^17^, ^18^, ^19^, ^20^, ^21^, ^22^ All the above have been linked to cancer (reviewed by Zhou, 2018),^2^ neurodegenerative disorders,^23^ cardiac disease (reviewed by Kandala, 2014),^24^ and others.

Notably, neddylation has emerged as a critical mechanism in regulating the immune system. Abnormal activation of neddylation pathway affects the proliferation, effector function, and signal transduction of diverse immune cells, leading to ectopic immune responses in vitro and in vivo. Accumulated evidence has proven that pharmacological intervention by MLN4924 and genetic deletion of key molecules in neddylation modification are inseparably related to alleviated immune‐mediated diseases, which include infection (reviewed by Han, 2018),^25^ inflammation and tumor. Herein, we aim to deeply clarify the roles and underlying mechanisms of neddylation modification as a regulator in the immune system. We also summarize various immune‐related diseases associated with neddylation dysregulation.

### 1.1 Neddylation emerges as a regulator of immune cells

**Figure 1:**
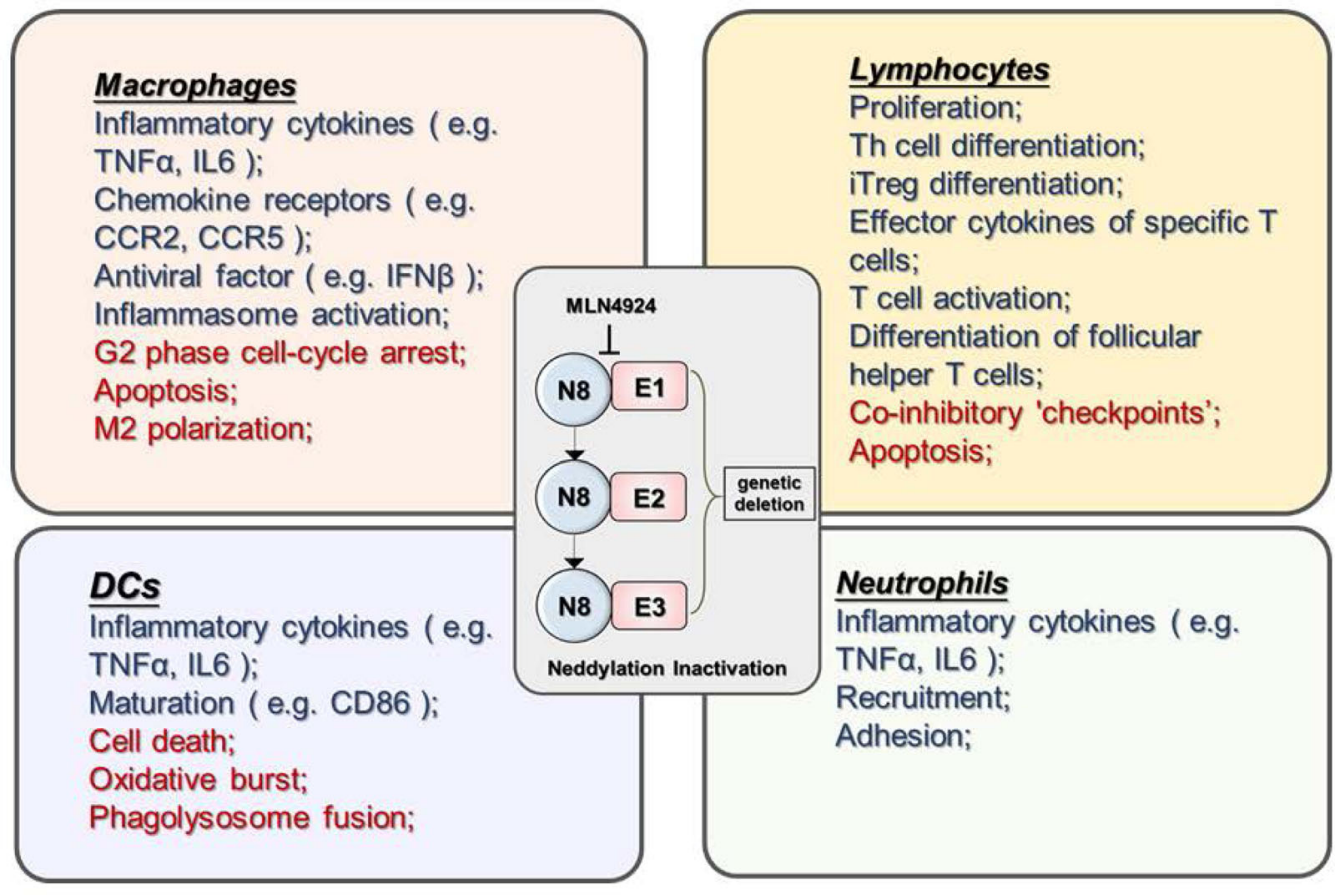
Neddylation inactivation by MLN4924 treatment or genetic deletion is involved in multiple regulatory responses within immune cells. Red, enhanced biological process by repressing neddylation modification; Blue, impaired biological process

Posttranslational protein modification is an essential influencing factor on innate and adaptive immune cells in aspects of survival, differentiation, recruitment, and effector function (Figure 1). In addition, it served in an indirect manner by modulating the crosstalk between immune cells and others.

### 1.2 Neddylation in innate immune cells

Neutrophils, a type of polymorphonuclear leukocyte, are recognized as the first line of defense in innate immune response who are able to be recruited to an inflammatory site to eliminate pathogens.^26^ Multiple mechanisms are involved in modulating biological roles of neutrophils, such as producing multifarious cytokines with wide functional diversity.^27^ Researches have shown that neddylation plays an important role in regulating neutrophils. Zhu et al^28^ found that deficiency of Cullin‐5, which belongs to the Cullin family that can form CRLs, attenuated alveolar neutrophil recruitment in lipopolysaccharide (LPS)‐challenged mice. In vitro study indicated that pharmacological agent MLN4924 led to decreased production of tumor necrosis factor‐α (TNF‐α), interleukin (IL)‐6, and IL‐1β in response to LPS in a dose‐dependent manner. Meanwhile, the viability of neutrophils was marginally affected.^29^ Intriguingly, Asare et al^30^ showed that MLN4924 treatment in vivo elevated neutrophil counts in blood. This phenotype may be caused by overcompensation for the early‐stage anti‐inflammatory effects of MLN4924 on endothelium. To sum up, neddylation modification acts as a crucial modulator of the neutrophil recruitment and effector function.

The “mononuclear phagocyte system” is identified as a population of originally bone marrow‐derived myeloid cells that circulate in the blood as monocytes and migrate to tissues as macrophages in a steady‐state and during inflammation.^31^, ^32^ It was demonstrated that blocking neddylation with MLN4924 or siRNA abrogated LPS‐induced proinflammatory cytokines (TNF‐α and IL‐6) secreted from murine and human macrophages.^33^, ^34^ The production of interferon‐β (IFN‐β), which is important for the elimination of viral infection, was also repressed in macrophage with MLN4924 treatment, whereas NEDD8 knockdown had no effects.^35^ Moreover, NEDD8 silencing or MLN4924 treatment led to diminished inflammasome activation and reduced IL‐1β maturation in macrophages.^36^ In a mouse model of bileductligation‐ or carbon tetrachloride‐induced fibrosis, neddylation inhibition was reported to reduce the expression of chemokine receptors and cytokines in Kupffer cells, the liver‐resident macrophages.^37^ Besides altered effector functions of macrophages, the viability of RAW264.7 was obviously impaired with persistent and severe inactivation of neddylation by MLN4924 while insignificant changed with partial inactivation.^34^ Due to the properties of the functional polarization and plasticity, macrophages are polarized into classically activated macrophages (M1) and alternatively activated macrophages (M2) in response to different signals.^38^, ^39^ Research indicated that MLN4924 drove macrophage polarization toward an anti‐inflammatory M2 phenotype even in the absence of exogenous polarizing signals.^30^ Collectively, neddylation modification is involved in controlling the polarization, the survival, and the inflammatory responses of macrophage.

Dendritic cells (DCs) are professional antigen‐presenting cells that bridge between innate and adaptive immunity. Beyond that, DCs also providing multiple soluble and surface‐bound signals so as to tailor distinct T‐cell differentiation programs.^40^, ^41^ Studies have proven that MLN4924 treatment, as well as siRNA‐mediated knockdown of RBX2/SAG in DCs, exhibited remarkable repression in the release of cytokines.^42^ Another research reported that inhibition of neddylation suppressed the release of proinflammatory cytokines by DCs but which was is not due to the result of decreased cell viability or phenotypic change.^43^ Notably, Mohamed El‐Mesery et al^44^ found that MLN4924 inhibited DC maturation by sensitizing immature DCs to TNF‐ and LPS‐induced necroptosis. Functionally, RNAi mediated knockdown of NEDD8 promoted the oxidative burst and phagolysosome fusion in mycobacteria infected DCs, as well as higher expression of autophagy and apoptosis‐associated proteins.^45^ However, current evidence demonstrated that the E3 ligase CRL4^DCAF2^ in DCs negatively regulated IL‐23 production.^46^ Thus, the immunoregulation of different neddylation components in DC inflammatory response remains elusive.

### 1.3 Neddylation in adaptive immune cells

T cells are central regulators of adaptive immune responses. Due to antigen stimulation, naïve T cells subsequently proliferate, differentiate and play their roles in connection to infection, cancer, autoimmunity disease, and alloreactivity.^47^ Jin et al^48^ have investigated the role of neddylation in regulating T‐cell function and demonstrated that deficiency of Ubc12 in CD4^+^ T cells resulted in diminished proliferation, Th1, and Th2 differentiation and cytokine production in vitro and in vivo. However, no obvious effect was observed on the development of CD4^+^, CD8^+^, or CD4^+^CD8^+^ thymocytes.^48^ SAG genetic knockout showed phenotypically normal mature T‐cell development and decreased activation, proliferation, and T‐effector cytokine release as well.^49^ The naïve T cells stimulated with various endogenous signals can polarize to different T‐cell subsets. Upon exposure to the NAE inhibitor, the proportion of Th1 cells moderately increased, whereas Th2 cells decreased.^50^ Meanwhile, NAE inhibition downregulates the differentiation of inducible Tregs.^50^ Apart from that, neddylation is also required for maintaining CD4^+^ T‐cell survival through repressed mitochondria‐dependent apoptosis.^51^ Tfh cells are a unique CD4^+^ T‐cell subset and are essential for the germinal center formation and effective humoral immunity.^52^ Evidence indicated that neddylation promotes Tfh cell differentiation via elevated forkhead box O1 (FoxO1) degradation in an Itch‐dependent manner.^51^ Coinhibitory “checkpoints,” such as PD‐1 and its ligand PD‐L1, are considered to cause T‐cell exhaustion and terminally diminished adaptive immune response.^53^ It was reported that inhibition of Cullin‐3 activity by MLN4924 attenuated T‐cell killing through blocked PD‐L1 protein degradation.^54^ In view of intricate differentiation and function of adaptive immune cells, our knowledge of that of neddylation is far from being desired.

### 1.4 Neddylation in other cells

Neddylation is also considered to regulate substrate proteins in other types of cells such as endothelial cells or epithelial cells, and their interaction with immune cells is influenced as a consequence. The state‐of‐art research has proven that treatment of human umbilical vein endothelial cells with a COP9 signalosome inhibitor (pharmacological activation of CRLs) enhanced intercellular cell adhesion molecule (ICAM) expression and resulted in consequent adhesion of neutrophils to endothelial cells.^55^ Moreover, the glioma cells after MLN4924 treatment were reported to have a stronger potential to induce T‐cell anergy.^56^ Recently, our colleagues uncovered that neddylation inactivation in tumor cell increased C‐C motif chemokine ligand 2 (CCL2) secretion and then promoted tumor‐associated macrophage infiltration.^57^

## 2 NEDDYLATION‐MODULATED SIGNALING MOLECULE IN THE IMMUNE RESPONSE

### 2.1 Neddylation and NF‐κB signal

Nuclear factor‐kappa B (NF‐κB) exists in most cell types and serves as a core regulatory molecular in the inducible expression of many proteins in innate and adaptive immune responses, including cytokines, cell adhesion molecules, and acute phase response proteins.^58^, ^59^ There are two types of NF­κB signaling pathways: the classical and the alternative pathway. The classical pathway is rapidly and transiently activated by pro­inflammatory cytokines, pathogen‐associated molecular patterns, and damage‐associated molecular patterns. The translocation of NF­κB dimers from the cytoplasm to the nucleus depends on the proteasomal degradation of the inhibitor of nuclear factor kappa B (IκB), following the phosphorylation by IKK complex.^60^, ^61^ Recent studies have highlighted our current knowledge of the posttranslational modifications in this signaling pathway. Studies have provided that the NEDD8 conjugation of Cullin‐1 has profound effects on the signal‐dependent degradation of IκBα.^62^, ^63^ Its polyubiquitination is triggered by the activation of SKP1‐CUL1/RBX1‐βTrCP CRL (SCF^βTrCP^), which is composed of cullin‐1 and RBX1 (the enzymatic core), SKP1 (an adaptor) and βTrCP (the substrate‐binding F‐box protein).^64^ Deneddylation removes the small protein NEDD8 from CRLs to the accumulation of IκB, which inactivating NF‐κB. The pharmacological blockade of the entire neddylation pathway by MLN4924 has been proven to cause accumulated IκB and stabilized NF‐κB as well. In a consequence, typical targets for NF‐κB such as pro­inflammatory cytokines (TNF‐α, IL‐6), chemokines (CCL2/MCP1), antirival factor (IFN‐β) and adhesion molecules (ICAM) are downregulated.^25^, ^55^, ^57^, ^65^ However, what's puzzling is that neddylation inactivation usually promotes the accumulation of phosphorylated IκBα, rather than total IκBα, in some studies.^34^, ^66^ It may be interpreted that other protein degradation pathways are involved in the regulation.

Few physiologic regulatory factors have been demonstrated to function on neddylation components so as to modulate the NF­κB pathway. The observations indicated that the interaction of nonpathogenic commensal bacteria with epithelial cells blocked the neddylation of the Culllin‐1 subunit of E3‐SCF^βTrCP^. Bacteria‐derived butyrate and other short‐chain fatty acids also rapidly induced the generation of reactive oxygen species and caused oxidative inactivation of Ubc12, resulting in attenuation of NF­κB activation.^67^, ^68^, ^69^ Besides, Glutamine administration could enhance Cullin‐1 neddylation and attenuate NEDD8 expression, which contributes to decreased NF­κB activation.^70^ It has been revealed that adenosine could modulate Cullin‐1 neddylation and then inhibit NF‐κB through modulating proteasomal degradation of IκB proteins.^71^ Additionally, Nedd8 is identified as a novel TRIM40‐binding protein. TRIM40 enhances neddylation of IKKγ and inhibits the NF­κB‐mediated transcription.^72^ The funny thing is that MyD88, a novel substrate of NEDD8, was thought to antagonize its ubiquitination and dimerization, and negatively regulate MyD88‐dependent NF‐κB signaling.^73^

### 2.2 Neddylation and HIF‐1α signal

Hypoxia‐inducible factor 1 (HIF‐1), a heterodimer composed of an oxygen‐regulated HIF‐1α subunit and a constitutively expressed HIF‐1β subunit,^74^ usually acts as a signaling hub to coordinate the activities of many transcription factors and signaling molecules.^75^ Under normoxic conditions, HIF‐1α is hydroxylated by prolyl hydroxylase domain proteins. Hydroxylated HIF‐1α can interact with the VHL protein, a substrate recognition subunit of E3 ubiquitin ligases, for proteasomal degradation.^76^ It's worth noting that specific metabolic imbalances, such as bacterial lipopolysaccharide stimulation, are able to induce HIF‐1α accumulation in the case of normal oxygen levels.^77^ Under hypoxic conditions, such as sites of inflammation and tumor, hydroxylation is repressed and HIF‐1α is stabilized and translocated to the nucleus for enhancing transcription of a number of inflammatory genes.^78^

HIF‐1α is also tightly regulated in a posttranslational fashion. As early as 2003, it was reported that VHL was covalently conjugated by NEDD8, not requiring for the E3 activity to ubiquitylated degradation of HIF‐1α. However, expression of a cullin neddylation‐defective VHL protein mutant in renal clear‐cell carcinoma cells restored the regulation of HIF‐1α,^11^ indicating mysterious neddylation modification of HIF‐1α. Sufan RI et al discovered that VHL triggered Rbx1‐mediated of Cullin‐2 neddylation, which promotes the engagement of UbcH5a to the ECV complex and subsequent recognition of HIF‐1α in an oxygen‐dependent manner.^79^ Interestingly, HIF‐1α substrate binding to ECV also was involved in promoting Cullin‐2 neddylation and mutation of HIF‐1α residues of VHL binding resulted in reduced Cullin‐2 neddylation.^80^ Additionally, NEDD8 covalently modified and stabilized HIF‐1 both in normoxia and hypoxia. Such an effect was diminished by antioxidants and mitochondrial respiratory chain blockers.^81^ The greatest attraction was that stress‐inducible UBE2M was *cis*‐transactivated by HIF‐1 and MLN4924 upregulated UBE2M via blocking degradation of HIF‐1α,^82^ indicating that HIF‐1α may also regulate transcription and degradation of neddylation components, not just as a substrate of neddylation.

Neddylation modulates various HIF‐1α‐transactivated effectors. MLN4924 was confirmed to repress transcriptional expression of ICAM‐1 and LPS‐induced endothelial permeability in mucosal inflammation. MLN4924 attenuated the transcription of a number of inflammatory cytokines, including IL‐1β, IL‐6, TNF‐α, IL‐12p70, and IFN‐γ. Additionally, an increase, albeit not statistically significant, in the anti‐inflammatory cytokine IL‐10 was also observed with MLN4924 treatment, which revealed an anti‐inflammatory effect of neddylation inactivation.^83^, ^84^ Targeting neddylation pathway contributed to a significant increase of HIF‐1α and PD‐L1 in gliomas and further resulted in T‐cell energy.^56^ Interestingly, most of the HIF‐1α‐transactivated inflammatory genes or other effect genes can be regulated by NF‐κB and HIF hydroxylases also regulate NF‐κB. In hypoxia inflammatory conditions, these two central transcription factors display an intimate interdependence via several mechanistic ways. Hence, the effects of neddylation on HIF‐1α and NF‐κB signaling should be more complicated and further balanced.^85^

### 2.3 Neddylation and other signals

Other than NF‐κB and HIF‐1α, little knowledge of neddylation‐targeted signals has been mentioned in regulating the immune response. Some CRL‐dependent and CRL‐independent substrates have already been identified, despite not being widely detected. Neddylation leads to repressed Deptor accumulation by the conjugation of Nedd8 to Cullin‐1 and sequentially promotes the mechanistic target of rapamycin kinase (mTOR) activity.^43^ Neddylation inactivation also upregulates PD‐L1 expression on glioblastoma cell lines by stabilization of c‐Myc.^54^ Adaptor protein Shc is another target for neddylation, which facilitating the formation of the ZAP70‐Shc‐Grb2 complex and downstream Erk activation.^48^ NEDD8 modification of Itch, leading to degradation of FoxO1, promotes Tfh cell differentiation.^51^ Treatment with MLN4924 contributes to increased Lipin‐2 protein stability by repressing interaction with Cullin‐1 and concomitantly decreased IL‐1β expression.^86^

## 3 NEDDYLATION EMERGES AS A REGULATOR OF IMMUNE‐MEDIATED DISEASES

### 3.1 Neddylation in infection

**Figure 2:**
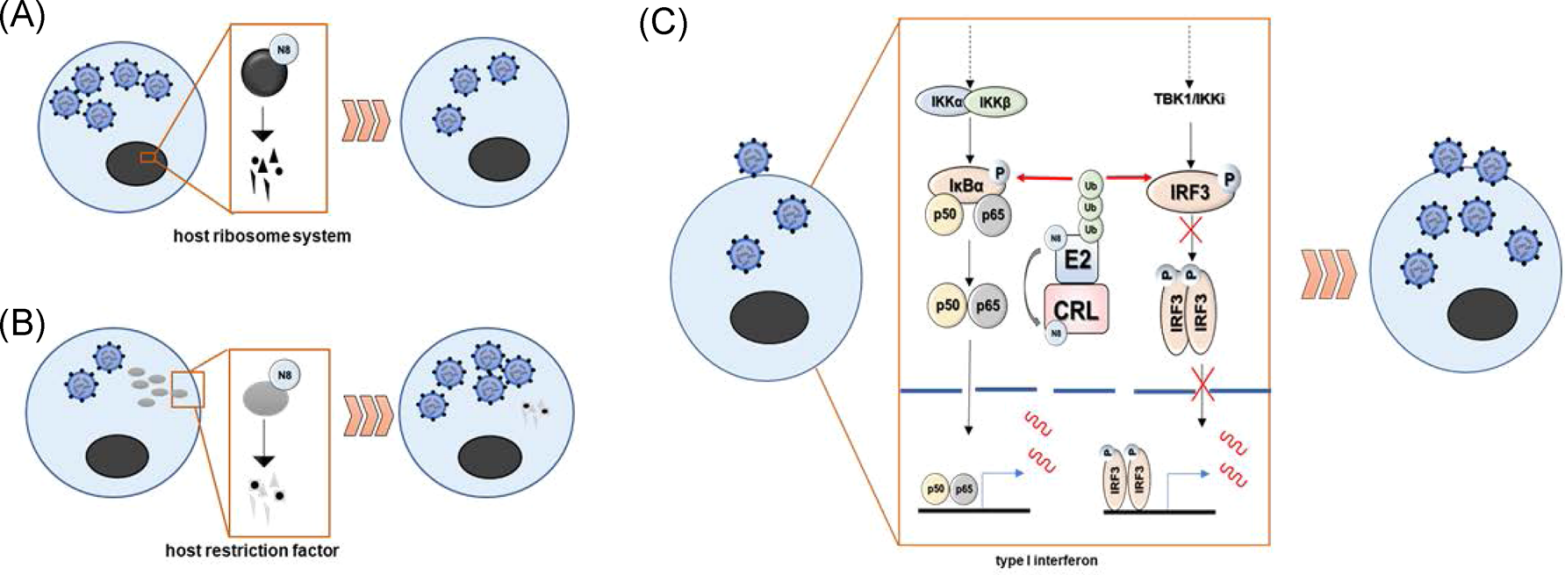
Mechanisms of neddylation modification against viral infection. A, Neddylation of proteins involved in the host ribosome system reduces its stability and blocks the replication of the virus. B, Neddylation modification contributes to the degradation of host restriction factors and releases suppression to the replication of the virus. C, Neddylation modulates the production of type I interferons mainly in NF‐κB‐ and IRF3‐dependent manner. Phosphorylated IRF3 or p50/p65 dimerizes and is translocated into the nuclear compartment to induce the expression of antiviral genes. Phosphorylated IRP‐3 and IκB are recognized by CRLs and induced the proteasomal degradation. CLR, Cullin‐RING E3 ligase; IRF, interferon regulatory factor; NF‐κB, nuclear factor kappa B

Infection is referred to a process that bacteria, viruses, fungi, or other pathogens enter the body and the subsequent biological changes. By far, the immunomodulatory effects of neddylation inhibition against viral infections have been concerned (Figure 2). First, neddylation is considered to control the amplification and replication of viruses. It is reported that blocking or silencing neddylation dramatically increased spring viremia of carp virus (an ssRNA virus that causes an important disease affecting cyprinids) replication.^87^ Zhang et al^88^ reported that neddylation of the polymerase basic protein 2 of influenza A virus reduces its stability and blocks the replication of influenza A virus. On the other hand, neddylation inhibition restores the restriction of human immunodeficiency virus by repressing the degradation of host restriction factors, a kind of cellular proteins inhibiting the replication of viruses at various stages of their life cycle.^89^, ^90^ Intriguingly, activated neddylation modification pathway led to increased virus growth in influenza A virus‐infected A549 cells.^91^ Hence, the modulatory effects of neddylation on amplification and replication may be disparate, depending on the virus species. Second, neddylation prevents viruses against immune‐evasive activities by controlling the production of type I interferon. It is demonstrated that neddylation inhibition with MLN4924 or upon UBA3 deficiency led to impaired IκBα degradation and NF‐κB‐promoted production of type I interferon in the early phase of herpes simplex virus type I (HSV‐1) infection, rather than the late phage, whereas no obvious effect of interferon regulatory factor (IRF) on type I interferon.^92^ The transcription of type I interferon genes is also mediated by IRF family members. Neddylation inhibition also results in the stabilization of IRF3 and production of type I interferon following Sendai virus infection.^93^ More interestingly, NEDD8 knockdown exhibits no effect on type I interferon production triggered by poly (I:C) or Sendai virus, which is reported to induce IRF3 degradation, but HSV‐1 does not.^93^ Hence, the modulatory effects of neddylation on the production of type I interferon may be determined by the interplay between the IRF and NF‐κB signal. Besides viruses, Cheng et al^51^ have described a crucial role of neddylation during the primary blood‐stage *Plasmodium* infection, in controlling the CD4^+^ T‐cell responses and subsequent humoral immunity.

### 3.2 Neddylation in inflammatory disease

Inflammation is considered as a defense mechanism that triggered by damage to the body and neddylation inactivation by genetic silencing or the specific inhibitor MLN4924 usually exhibits beneficial effects on the disease control. For LPS‐induced inflammatory responses such as acute kidney injury and acute lung injury, inactivation of neddylation has been demonstrated to inhibit the CRL/NF‐κB signal mediated production of proinflammatory cytokines (eg, TNF‐α and IL‐6) so as to alleviate the inflammation.^28^, ^65^ Additionally, diminished neddylation activity via MLN4924 could be important for resolving liver fibrosis and pulmonary fibrosis through reduced expression of the chemokines from epithelial cells and subsequently impaired induction of chemokine receptors and cytokines in activated macrophages.^37^, ^94^ Mice transferred with Ubc12 knockdown CD4^+^ T cells exhibited a deficiency in the ability to develop Th2‐mediated allergic response in OVA‐driven airway inflammation.^48^ The immunomodulation of neddylation inhibition on mucosal inflammation has also been described, indicating a potential therapeutic opportunity in inflammatory bowel diseases. Neddylation inhibition suppressed CRL/Deptor‐mediated mTOR signaling pathway in DCs.^43^ Meanwhile, inhibition of the neddylation is capable of enhancing barrier function of intestinal epithelial cells in a CRL/HIF‐dependent way, which may be disturbed by DEN‐1.^84^ However, neddylation inhibition by MLN4924 treatment combined with proinflammatory cytokines may lead to increased epithelial CRL/NF‐κB‐mediated apoptosis and barrier disruption,^95^ indicating that MLN4924 treatment in inflammatory conditions may cause side effects as well as more aggravated diseases.

### 3.3 Neddylation in cancer

A blend of epithelial cells, endothelial cells, stromal cells (eg, immunocytes and fibroblast cells), and soluble factors interplay with each other and constitute the tumor immune microenvironment.^96^ Neddylation inactivation, as well as MLN4924 (also known as pevonedistat, currently in phase I/II clinical trials for patients suffering from cancer), have been proven to exert anti‐tumor activity by modulating cellular processes in tumor cells, but their influences on the functions of immunocytes in the tumor microenvironment are rarely studied. Zhou L et al^57^ discover for the first time to our knowledge that, neddylation inactivation exhibits its anti‐tumor activity by suppressing the recruitment of monocytes/tumor‐associated macrophages in the tumor microenvironment, in response to Cullin‐1/NF‐κB‐modulated CCL2 derived from tumor cells.^57^ Another study indicated that neddylation inhibition significantly enhances Cullin‐1/c‐Myc‐modulated PD‐L1 expression in glioblastoma cancer cell lines, resulting in T‐cell exhaustion and attenuated T‐cell killing.^54^ Besides of solid tumors, unique effects of neddylation inhibition on lymphoid malignancies have been discussed recently. NAE inhibition treated T cells derived from patients with chronic lymphocytic leukemia exert markedly differential expression of NF‐κB‐regulated genes and downregulated of interleukin‐2 signaling during T‐cell activation. In addition, NAE inhibition causes decreased Treg differentiation and a shift toward the Th1 phenotype, accompanied by increased interferon‐γ production.^50^ Altogether, the neddylation pathway is capable of modulating the immune microenvironment in tumors via diverse ways, but much more remains to be concerned.

## 4 CONCLUDING REMARKS

Neddylation modification was originally identified as a posttranslational regulator that highly conserved exists in a variety of cell types and species. New insights into regulatory roles for neddylation in immune responses have come of interest but efforts will still be made to intensify the investigations in this field. The existing investigations highlight that neddylation pathway is engaged in many aspects of the immune cell biology such as survival, differentiation, and immune effector function. Abnormal neddylation alters the characteristics and functions of immune cells and further plays pivotal roles in different stages of immune‐related diseases. Additionally, the following aspects of the immunology of neddylation modification need to be concerned.

First, most of our knowledge about the role of neddylation in various processes of immune responses depends on MLN4924, a potent and selective inhibitor which blocks the first cascade of neddylation pathway by competitively binding to NAEβ. By far, few inhibitors targeting other components of neddylation pathway exist. It would be fascinating whether other targeting inhibitors of neddylation pathway would show effective modulation as well as disparate effects compared with MLN4924 on immune responses. More selective targeting inhibitors effective on immunoregulation will kindle a spark of hope in clinical application.

Second, neddylation modification is extremely well conserved and NEDD8 is ubiquitously expressed in all cell types in the body.^3^ Considering that MLN4924 treatment gives rise to global inactivation of neddylation, it may be difficult to clarify the detailed mechanisms for intricate diseases in vivo. Hence, conditional knockout mice that lack specific components of neddylation pathway are in demand. Previous research has directly elucidated the effects of neddylation on T‐cell mediated parasite control and host survival against *Plasmodium* infection by crossing the *Uba3* ^*fl/fl*^ mice with the *Lck‐Cre* transgeneic strain to generate *Uba3* ^*fl/fl*^ *Lck‐Cre* ^*+*^ mice, in which neddylation inhibition is confined to the T‐cell compartment.^89^ Likewise, despite of numerous researches of neddylation acting on tumor cells, the regulation of other components in the microenvironment altered by neddylation modification could be investigated by crossing flox mice with *LysM‐Cre* transgenic strain, *Fsp1‐Cre* transgenic strain and so on.

Third, little is known about the immunoregulation of neddylation on chronic inflammation and inflammation‐associated tumorigenesis which undergo a long time of progression. It is reported that p38α negatively regulates the initiation of colitis‐associated colon tumors, whereas transformed intestinal epithelial cells rely on p38α signaling for survival and proliferation,^97^ indicating a dual function of p38α signaling in colitis‐associated cancer. Then, dose neddylation inhibition induced in different stages of the disease progression exhibit disparate function? An in vivo research demonstrated that MLN4924 treatment decreased the progression of early atherosclerotic lesions in mice, but had no net effect on the progression of more advanced lesions. In contrast to inflammation elimination in the early stage, MLN4924 treatment exhibited an increased level in neutrophil and monocyte counts in blood in the advanced stage,^30^ suggesting discrepant and complicated roles of neddylation in chronic (ongoing) diseases. Notably, the inducible CreERT2 transgenic mice, in which the Cre activity is only detected in the presence of tamoxifen,^98^ are considered to be appropriate for such investigations.

In a word, the investigation we have summarized here deepens our understanding of the role of neddylation pathway in fundamental immunology which help to unveil the pathogenesis of immune‐related diseases, and provides a foundation for neddylation‐based therapies in disease treatment.

## CONFLICT OF INTERESTS

The authors declare that there are no conflict of interests.

## Data Availability

All data are shown within the manuscript and figures.
